# Killer yeasts exert anti-plasmodial activities against the malaria parasite *Plasmodium berghei* in the vector mosquito *Anopheles stephensi* and in mice

**DOI:** 10.1186/s13071-019-3587-4

**Published:** 2019-07-02

**Authors:** Alessia Cappelli, Matteo Valzano, Valentina Cecarini, Jovana Bozic, Paolo Rossi, Priscilla Mensah, Consuelo Amantini, Guido Favia, Irene Ricci

**Affiliations:** 1 School of Biosciences and Veterinary Medicine, University of Camerino, Camerino, Italy; 2 Florida Medical Entomology Laboratory, University of Florida, Vero Beach, FL USA

**Keywords:** *Wickerhamomyces anomalus*, *Plasmodium berghei*, *Anopheles stephensi*, Killer toxin, Symbiotic control, Malaria

## Abstract

**Background:**

*Wickerhamomyces anomalus* is a yeast associated with different insects including mosquitoes, where it is proposed to be involved in symbiotic relationships with hosts. Different symbiotic strains of *W. anomalus* display a killer phenotype mediated by protein toxins with broad-spectrum antimicrobial activities. In particular, a killer toxin purified from a *W. anomalus* strain (*Wa*F17.12), previously isolated from the malaria vector mosquito *Anopheles stephensi*, has shown strong *in vitro* anti-plasmodial activity against early sporogonic stages of the murine malaria parasite *Plasmodium berghei*.

**Results:**

Here, we provide evidence that *Wa*F17.12 cultures, properly stimulated to induce the expression of the killer toxin, can directly affect *in vitro P. berghei* early sporogonic stages, causing membrane damage and parasite death. Moreover, we demonstrated by *in vivo* studies that mosquito dietary supplementation with activated *Wa*F17.12 cells interfere with ookinete development in the midgut of *An. stephensi*. Besides the anti-sporogonic action of *Wa*F17.12, an inhibitory effect of purified *Wa*F17.12-killer toxin was observed on erythrocytic stages of *P. berghei*, with a consequent reduction of parasitaemia in mice. The preliminary safety tests on murine cell lines showed no side effects.

**Conclusions:**

Our findings demonstrate the anti-plasmodial activity of *Wa*F17.12 against different developmental stages of *P. berghei*. New studies on *P. falciparum* are needed to evaluate the use of killer yeasts as innovative tools in the symbiotic control of malaria.

**Electronic supplementary material:**

The online version of this article (10.1186/s13071-019-3587-4) contains supplementary material, which is available to authorized users.

## Background

Malaria is a mosquito-borne disease that kills about half a million people each year, predominantly children under five years of age. *Plasmodium*, the etiological agent of malaria, is transmitted to humans (vertebrate host) through the bite of a female *Anopheles* mosquito (vector and invertebrate host). Efforts to reduce morbidity and mortality have been impaired by drug and pesticide resistance and the lack of effective vaccines. To ameliorate the efficacy of malaria control efforts, innovative strategies are urgently needed [1, 2].

*Plasmodium* has a complex life-cycle including sexual and asexual developmental stages. It alternates a sporogonic phase in the mosquito with differentiation of zygotes, ookinetes, oocysts and sporozoites, and hepatic/erythrocytic phases in the vertebrate host where it multiplies different asexual stages, micro- and macro-gametocytes. This multifaceted phenotypic variability is advantageous for the parasite but hampers the development of new therapeutic drugs and/or prophylaxis, thus facilitating the spread of malaria.

In recent years, symbiotic control (SC) has been proposed as a possible strategy for preventing malaria [3]. SC is intended to block malaria transmission by reducing anopheline vector competence through symbiotic microbes that affect the development of the parasite in the mosquito midgut [4, 5]. Some bacteria, including *Asaia*, *Wolbachia* and *Pantoea*, and fungi such as *Metarhizium*, have been proposed for the SC of malaria [3, 6–8]. In this frame, the identification of killer yeasts that exert symbiotic functions in mosquitoes is very promising [9, 10].

Among the different isolated symbiotic killer strains, *Wickerhamomyces anomalus* offers a series of highly appealing features [11]. In fact, *W. anomalus* displays a wide antimicrobial property mediated by killer toxins (KTs) that exert an enzymatic activity targeting the cell-wall glucan components of bacteria, yeasts and protozoa [12]. Interestingly, *W. anomalus* is considered safe [13] and some environmental strains are used against spoilage microbes for food and beverage bio-preservation [14, 15].

Symbiotic strains of *W. anomalus* producing KTs have been identified in beetles, mosquitoes and sand flies where they are proposed to be involved in nutritional and/or protective functions [16–18]. In particular, the *W. anomalus* strain (*Wa*F17.12) isolated from the malaria vector *Anopheles stephensi*, produces a KT (*Wa*F17.12-KT) that has shown, as a purified product, a strong anti-plasmodial activity *in vitro* against sporogonic stages of the murine malaria parasite *Plasmodium berghei*, causing a surface membrane damage mediated by a β-glucanase activity [19]. *Wa*F17.12-KT seems to be poorly expressed in the mosquito, although its production can be stimulated by selective media conditions, and yeast cultures can be reintroduced into the mosquito through diet [20]. Generally, the killer phenotype is activated or enhanced under stress conditions, as the competition with other microorganisms for environmental resources, whereas the host body is a niche favorable to the proliferation of yeast with good availability of food [21].

In the present study, *in vitro* and *in vivo* studies have been carried out to unveil interactions between the killer strain *Wa*F17.12 and the murine malaria parasite *P. berghei*. The results demonstrated that the yeast affects different stages of the parasite development by *Wa*F17.12-KT mediated mechanisms, both in mosquitoes and mice (vertebrate host). These outcomes validate *Wa*F17.12 as an effective tool against *P. berghei*.

## Methods

### Yeasts and killer toxin production

Two *W. anomalus* (*Wa*) strains were used in the present study: *Wa*F17.12, isolated from *An. stephensi* mosquitoes (KT-producer) [17]; and *Wa*UM3, environmental strain (KT non-producer) [22]. Both strains were cultured in selective growth conditions to stimulate KT production: YPD broth (20 g/l peptone, 20 g/l glucose, 10 g/l yeast extract), buffered at 4.5 pH with 0.1 M citric acid and 0.2 M K_2_HPO_4_ [23]. Cells were incubated at 26 °C for 36 h at 70× *rpm*. To verify the activation of *Wa*F17.12 and confirm KT expression, the supernatants of both yeast cultures were separated by chromatography and the obtained fractions analysed by western blot using the monoclonal mAbKT4 antibody specific for yeast KTs, as described in Valzano et al. [19] and Cappelli et al. [20]. The positive fraction obtained from *Wa*F17.12 was named as *Wa*F17.12-KT^+^ and the corresponding fraction isolated from *Wa*UM3 (*Wa*F17.12-KT^-^) was used as KT negative control.

### Malaria parasites

Two *P. berghei* (murine malaria parasite) transgenic strains were used in the present work: *Pb*CTRPp.GFP that expresses green fluorescent protein (GFP) during the early sporogonic phase of the parasite cycle [24], and *Pb*GFP_CON_ that expresses GFP throughout the whole parasite cycles both in the mosquito and vertebrate host [25]. The two parasites were differently used, depending on the type of assay. *Pb*CTRPp.GFP was used for cultivating early sporogonic stages *in vitro*, whereas *Pb*GFP_CON,_ which is detectable during all the developmental stages, was used during *in vivo* experimentations. Both parasite strains were visualized using a fluorescence microscope (Axio Observer.Z1; Carl Zeiss, Milan, Italy).

### Mice

BALB/c mice were maintained in the Animal Facilities of the University of Camerino at 24 °C, fed on standard laboratory mice pellets (Mucedola S.r.l., Milano, Italy) and provided with tap water *ad libitum*. Mice were used as *P. berghei* vertebrate hosts. Eight-week-old female mice (18–25 g) were infected with *Pb*CTRPp.GFP or *Pb*GFP_CON_ directly by an intraperitoneal injection of 10% parasitaemic blood obtained from the tail of a donor mouse (acyclic passage). About 10^6^ infected erythrocytes of the donor mouse were diluted in 200 μl of PBS (7.2 pH) and parasitaemia in injected mice was constantly monitored by optical microscope using a 10% Giemsa stained blood smear and fluorescence microscope (Axio Observer.Z1). Recipient mice were used for experimental purposes when showing 5% parasitaemia. All animal rearing and handling was carried out according to the Italian Legislative Decree (116 of 10/27/92) on the “use and protection of laboratory animals” and in agreement with the European Directive 2010/63/UE. The experimentation was approved by the Ethical Committee of University of Camerino.

### Mosquitoes

*Anopheles stephensi* (Liston strain) mosquitoes were reared at 29 °C and 85–90% relative humidity with a 12:12 light-dark photoperiod in the insectarium of the University of Camerino. Immediately after emergence of adult mosquitoes, three cages, each containing 250 females, were set up for two trials. Newly emerged mosquitoes were fed *ad libitum* on a cotton pad soaked with 5% (w/v) sterile sucrose solution (control group), or 5% sucrose solution plus *Wa*F17.12, or 5% sucrose solution plus *Wa*UM3.

### Killing activity assay of *Wa*F17.12 against *P. bergheiPb* CTRPp.GFP sporogonic stages *in vitro*

Activated *Wa*F17.12 and *Wa*UM3 cultures were pelleted at 3000× *g* for 10 min and suspended in PBS (7.4 pH), whereas the supernatants were processed as described above. *Pb*CTRPp.GFP cultures were obtained as follows: early sporogonic stages were developed *in vitro* incubating gametocytemic blood (20 µl) from a donor mouse showing 5% parasitaemia with ookinete-medium (180 µl) on microtiter plates. Ookinete-medium was prepared as described in Valzano et al. [19]. For the killing activity assay, parasite cultures were incubated with activated *Wa*F17.12 (20 μl) or *Wa*UM3 (20 μl) (yeasts final concentration 10^8^ cell/ml) for 24 h at 19 °C in the dark (20 μl of PBS 7.4 pH was added to control wells). During incubation, gametocytes develop into fluorescent zygotes and ookinetes. The parasite number in each well was evaluated using a fluorescence microscope with a 40× objective (Axio Observer.Z1). Experiments were performed in triplicate.

### Cell viability assay of *P. berghei* (*Pb*CTRPp.GFP)

*Wa*F17.12-KT^+^ and *Wa*UM3-KT^−^ fractions were tested *in vitro* against *Pb*CTRPp.GFP early sporogonic stages to prove effective activation of yeasts and KT production. A viability assay was carried out incubating gametocytaemic blood with ookinete-medium and *Wa*F17.12-KT^+^ (100 µg/ml KT final concentration) for 24 h at 19 °C. *Wa*UM3-KT^−^ was tested to evaluate possible effects due to other co-purified molecules, whereas control wells were prepared by adding PBS (7.4 pH). After incubation, parasites were treated with 20 µg/ml of the red-fluorescent dye propidium iodide (PI) (Sigma-Aldrich, Saint Louis, USA) in DNAse-free PBS, for 30 min at 20 °C, and visualized by fluorescence microscope (Axio Observer.Z1; Carl Zeiss, Milan, Italy). PI staining is used for identifying dead or damaged cells, because the dye penetrates cells with altered membranes where it intercalates DNA double strand, but it is effectively excluded from viable cells.

### Killing activity assay of *Wa*F17.12 against *P. berghei Pb*GFP_CON_ sporogonic stages *in vivo*

Three cages, each containing newly emerged female mosquitoes (*n* = 250), were set up with a cotton pad soaked with 5% sterile sucrose solution (control group), 5% sucrose solution plus *Wa*F17.12 or 5% sucrose solution plus *Wa*UM3. Yeasts were grown under stimulating conditions to produce KT as described above. Yeast cultures were centrifuged at 3000× *g* for 10 min at 4 °C, washed three times in 0.9% (w/v) NaCl solution, and suspended in 5% sucrose solution at a final concentration of 10^8^ cells/ml. Food preparations were provided *ad libitum* to mosquitoes and the cotton pad was refreshed every two days for the duration of the experiment (21 days). To verify the presence of activated yeasts in the mosquito gut, an immunofluorescent assay (IFA) assay at 24 h, and 10 and 21 days post-infection was performed using a monoclonal anti-KT antibody (mAbKT4) obtained at a concentration of 2 mg/ml, as described by Cappelli et al. [20]. After six days, mosquitoes had a blood meal on a 5% parasitaemic mouse infected with *Pb*GFP_CON_. Unfed mosquitoes were removed and cages were laid in a chamber at 19 °C and 95 ± 5% relative humidity for *P. berghei* development in the mosquito. Twenty-four hours after the blood meal (before ookinetes migrate across the midgut epithelium [26]), mosquito guts (*n* = 24 per cage) were dissected and individually homogenised in sterile PBS. Gut preparations were analysed through fluorescence microscopy using a 100× objective (Axio Observer.Z1) to detect *Pb*GFP_CON_ early sporogonic stages. The number of oocysts and sporozoites was evaluated, analysing guts and salivary glands (*n* = 10 per cage) 10 and 21 days post-infection, respectively. Experiments were performed twice.

### *Wa*F17.12-KT^+^ anti-plasmodial activity against *P. berghei Pb*GFP_CON_ erythrocytic stages in mice

A *Pb*GFP_CON_ infected mouse showing 5% parasitaemia was used for testing KT effects against *P. berghei *erythrocitic stages. The infected blood (100 µl) was incubated for 90 min at 25 °C with *Wa*F17.12-KT^+^ (100 µg/ml KT), *Wa*F17.12-KT^+^ (100 µg/ml KT) and mAbKT4 (100 µg/ml) [22], or 1× PBS at 7.2 pH (control). Preparations of treated blood samples (10^6^ infected red blood cells) were inoculated into healthy eight-week-old female mice (18–25 g) (*n* = 5 mice per group). After 5 days, the parasitaemia of recipient mice was evaluated using a 10% Giemsa stained blood smear and optical microscope with 100× objective. The parasitaemia was controlled in the three groups, and variation was calculated as reported in Bonkian et al. [27]. The experiment was repeated twice.

### Colorimetric MTT assay and Hepa 1–6 cellspropidium iodide (PI) staining

The colorimetric MTT assay was used to evaluate the cell viability in murine cell lines after treatment with *Wa*F17.12-KT^+^. Hepa 1–6 cell line (mouse/liver/hepatoma from American Type Culture Collection, Manassas, USA) were cultured at 37 °C in a humidified atmosphere of 5% CO_2_ using as a basal medium Dulbecco’s Modified Eagle’s Medium supplemented with 10% (v/v) heat-inactivated fetal bovine serum, 2 mM l-glutamine, 100 IU/ml of penicillin and 100 μg of streptomycin (Lonza, Switzerland). Hepa 1–6 cells/ml (4 × 10^5^) were seeded into 96-well plates and cultured with different doses of *Wa*F17.12-KT^+^ (1, 5, 10, 20, 40, 60, 80 and 100 µg/ml KT) for 24 h. Upon treatment, MTT (0.8 mg/ml) was added to the samples and incubated for 3 h. Then, the supernatants were discarded, and coloured formazan crystals dissolved with DMSO (100 μl/well) were read by an enzyme-linked immunosorbent assay reader (BioTek Instruments, Winooski, VT, USA). Four replicates were used for each treatment. Hepa 1–6 cells, treated with 100 μg/ml KT or relative vehicle for 24 h were incubated with 2 μg/ml PI for 10 min at 37°C. Then, cells were harvested with Versene (EDTA), washed and analyzed using a FACScan cytofluorimeter with CellQuest software.

### Statistical analysis

Statistical analyses were performed using GraphPad Prism 5 software. The differences between the groups in each experiment were compared using a Mann–Whitney U-test. A *P*-value < 0.05 was considered statistically significant.

## Results

### *Wa*F17.12 affects sporogonic stages of *P. berghei in vitro* and *in vivo*

The anti-plasmodial activity of *Wa*F17.12-KT has been demonstrated *in vitro* against cultivated *P. berghei* sporogonic stages, but any direct effect of such KT-producing yeasts on the parasite development still required validation. Therefore, in the present study, *in vitro* and *in vivo* experiments were carried out to evaluate direct interactions between *Wa*F17.12 and the murine malaria parasite *P. berghei*.

**Fig. 1:**
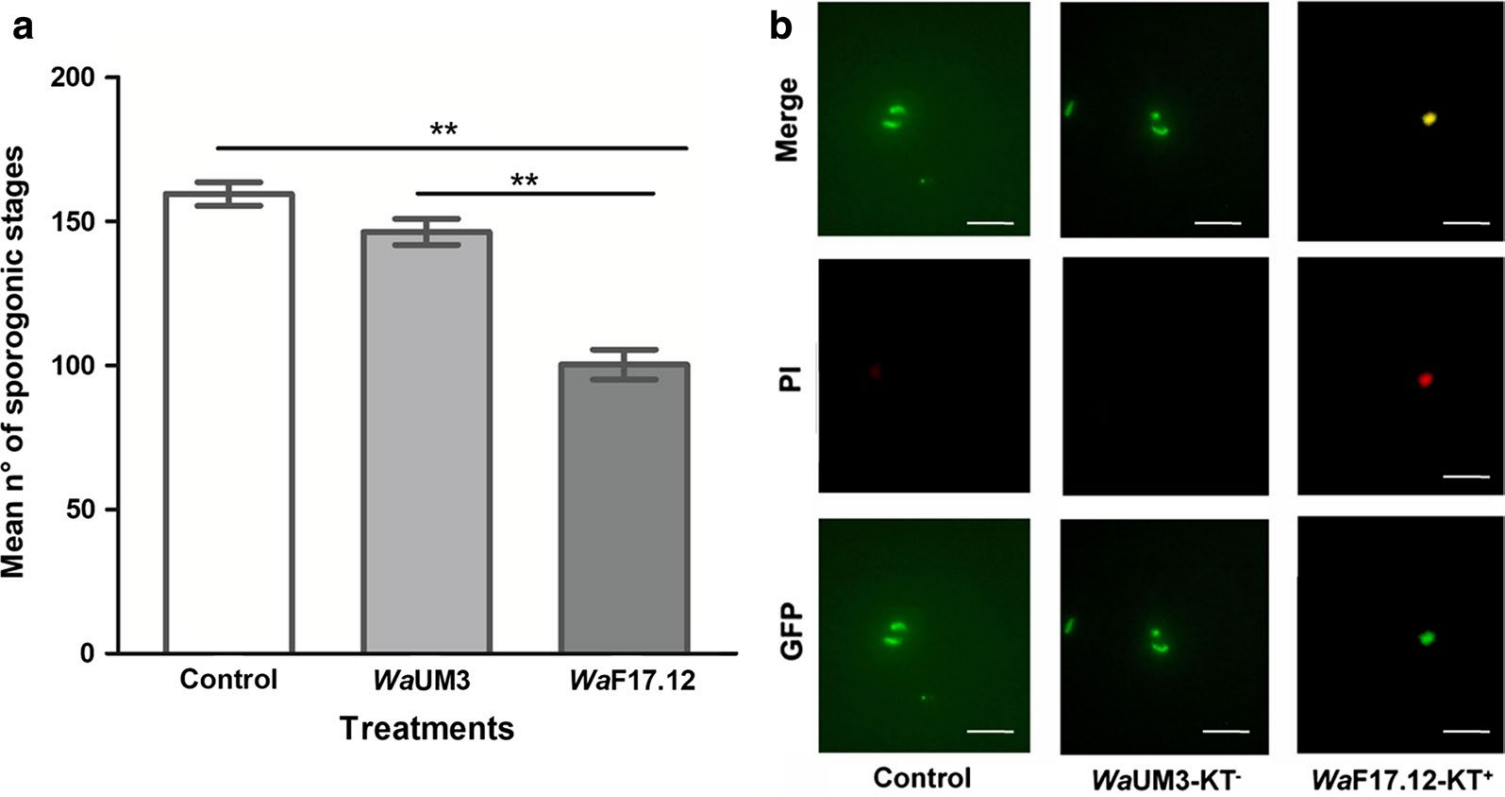
*Wa*F17.12 *in vitro* killing activity against sporogonic stages of *Pb*CTRPp.GFP (**a**) and cell viability assay (**b**). **a** Numbers of parasites developed by cultural method from gametocytemic mice bloodare reported after 24 h of incubation at 19 °C in the presence of *Wa*UM3 (10^8^ yeast cells/ml), *Wa*F17.12 (10^8^ yeast cells/ml) and PBS (control group). Mean values ± SEM were estimated from triplicate experiments and statistical significance expressed as a *P*-value (Mann–Whitney test: Control *vs Wa*UM3: *U*_(9)_ = 81, *Z* = 4.101, *P* = 0.0041; Control *vs Wa*F17.12: *U*_(9)_ = 81, *Z* = 4.101, *P* = 0.0041). **P < 0.01. **b** Fluorescence microscope images of ookinetes treated with the *Wa*F17.12-KT^+^ (containing 100 µg/ml KT) or *Wa*UM3-KT^−^ fraction. Data show an abnormal morphology and PI accumulation only in toxin-treated parasites. *Scale-bars*: 20 µm

During *in vitro* tests, the number of developed parasites (zygotes and ookinetes) was counted and the mean values ± SEM from triplicate experiments are reported for each group (Fig. 1a). The treatment with *Wa*F17.12 decreased the number of parasites by approximately 37.1%, whereas no significant differences between *Wa*UM3 and control were detected. Such data suggests that the effect of *W. anomalus* on *P. berghei* sporogonic stages development was strain-dependent and attributable to the presence of the KT in the medium. To assess the activation of the *Wa*F17.12 strain and to confirm KT expression, the supernatants from both strains were separated by ion-exchange chromatography and obtained fractions were analysed by western blot as described in the material and methods section. The KT containing fraction (*Wa*F17.12-KT^+^) and the corresponding KT^−^ (*Wa*UM3-KT^−^) were collected and tested against cultured early *P. berghei* sporogonic stages (Fig. 1b). A cell viability assay was performed using the propidium iodide (PI) dye, to evaluate the effect of individual purified fractions on parasite development. Treatment with *Wa*F17.12-KT^+^ induced an alteration of ookinetes morphology, characterized by bigger and more jagged shapes, and PI penetration in damaged and non-viable parasites. Conversely, *Wa*UM3-KT^−^ did not affect ookinetes, which showed a normal shape and no PI accumulation, similar to the control group, demonstrating that the anti-plasmodial activity of the *Wa*F17.12-KT^+^ fraction was specific and not due to the presence of other co-purified molecules.

**Fig. 2:**
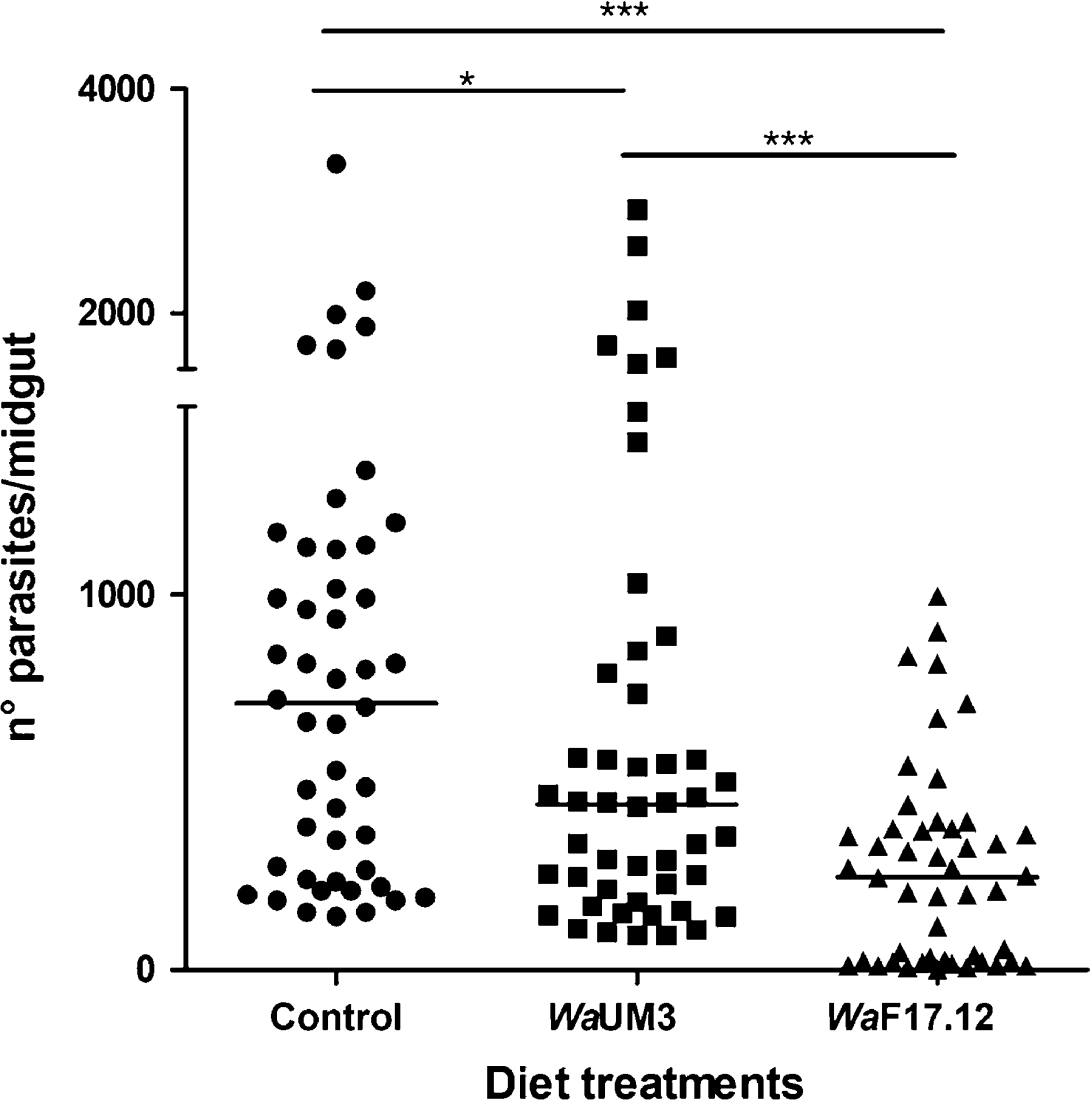
*Wa*F17.12 inhibitory effect on early sporogonic stages of *Pb*GFP_CON_ in *An. stephensi*. Number of parasites (zygotes and ookinetes) in the midgut of mosquitoes fed with sugar solution supplemented with *Wa*F17.12 (triangles), *Wa*UM3 (squares) or sugar solution (dots) was evaluated at 24 h post-infection. Each symbol in the chart represent an individual midgut (*n* = 48 per group) and horizontal lines represent the medians (m) of two independent experiments (m = 710 for control group, m = 440 for *Wa*UM3 group and m = 247 for *Wa*F17.12 group). Statistical analysis was performed by multiple comparisons using the Mann–Whitney test (Control *vs Wa*UM3: *U*_(48)_ = 882.5, *Z* = 1.971, *P* = 0.04884; Control *vs Wa*F17.12: *U*_(48)_ = 486.5, *Z* = 4.87287, *P *< 0.0001; *Wa*UM3 *vs Wa*F17.12: *U*_(48)_ = 684, *Z* = 3.42567, *P* = 0.0006). *P < 0.05, ***P < 0.001

These findings prompted additional *in vivo* studies on the evaluation of the *Wa*F17.12 strain anti-plasmodial activity in *An. stephensi*. The anti-plasmodial activity results confirmed the ability of *Wa*F17.12 to abundantly colonize the *An. stephensi* gut, whereas no KT signal was detected in mosquitoes treated with *Wa*UM3 and in the control group. *Pb*GFP_CON_ early sporogonic stages in the midgut lumen of mosquitoes colonized with activated yeast cultures and in control groups, were counted 24 h post-infection and the median values (m) obtained from two independent experiments are reported (Fig. 2). Mosquitoes colonized with *Wa*F17.12 developed 65.2% fewer parasites than the control group (m = 710 and m = 247, respectively) and 43.9% fewer parasites than the *Wa*UM3 group (m = 440). Differently from the *in vitro* analysis, a significant decrease in parasite number of 38.0% with respect to the control was also detected in mosquitoes colonized with *Wa*UM3, indicating a non-KT related anti-plasmodial activity of this strain.

Further analyses were then performed to evaluate possible inhibition effects of *P. berghei* (*Pb*GFP_CON_) in the later stages of the sporogonic cycle. Specifically, guts and salivary glands of 10 mosquitoes for each group were analysed at 10 and 21 days post-infection, respectively. The number of oocysts and sporozoites was not significantly different in the three groups (data not shown). Nevertheless, the huge number of oocysts developing during experimental infections could have impaired the detection of anti-plasmodial effects in the later sporogonic stages.

### *Wa*F17.12-KT^+^ anti-plasmodial activity against *P. berghei Pb*GFP_CON_ erythrocytic stages and preliminary safety tests

**Fig. 3:**
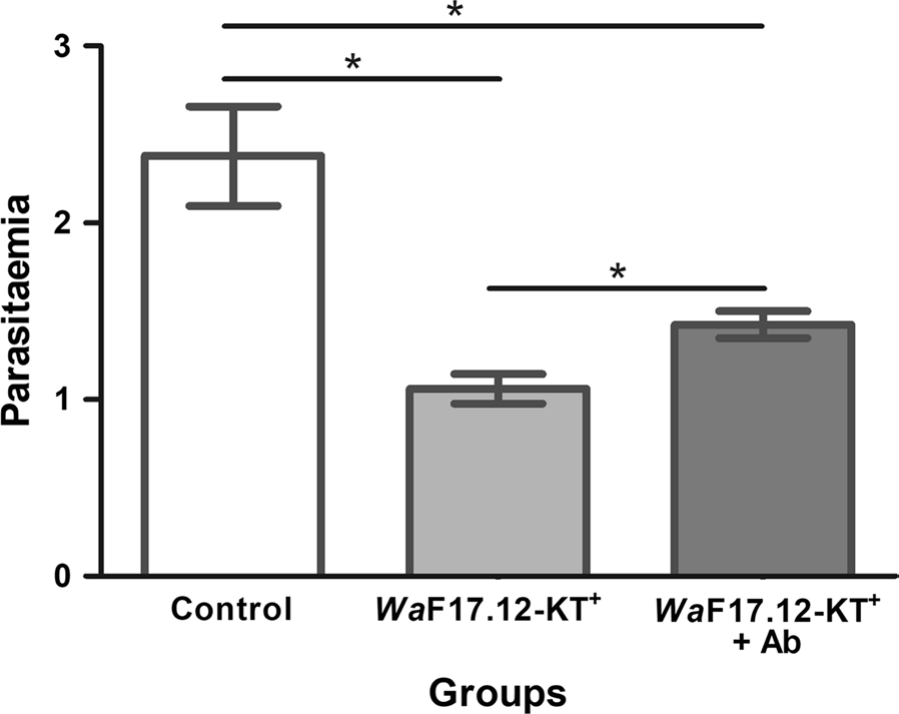
*Wa*F17.12-KT reduces parasitaemia in *Pb*GFP_CON_ infected mice. Healthy mice were inoculated using *Pb*GFP_CON_-infected blood of a donor mouse previously incubated for 90 min at 25 °C with *Wa*F17.12-KT^+^ (100 µg/ml KT), *Wa*F17.12-KT^+^ (100 µg/ml KT) and mAbKT4, or PBS (control). Five days after inoculations, parasitaemia of the recipient mice (*n* = 5 per group) was evaluated and mean values ± SEM, calculated as reported in Bonkian et al. [34], are reported. The experiment was repeated twice. Statistical analysis was performed using the Mann–Whitney test (Control *vs Wa*F17.12-KT^+^: *U*_(10)v_= 124, *Z* = 2.04228, *P* = 0.04136; Control *vs Wa*F17.12-KT^+^ + Ab: *U*_(10)_ = 83, *Z* = 1.790, *P* = 0.03673; *Wa*F17.12-KT^+^ *vs* Ab: *U*_(10)_ = 85, *Z* = 1.690, *P* = 0.03656). *P < 0.05

*Pb*GFP_CON_ infected donor mice were used to test the effects of *Wa*F17.12-KT on erythrocytic stages. Infected blood samples were individually incubated for 90 min at 25 °C with *Wa*F17.12-KT^+^ (100 µg/ml KT), or *Wa*F17.12-KT^+^ (100 µg/ml KT) plus the specific anti-KT antibody mAbKT4 (100 µg/ml) [22], or PBS at 7.2 pH (control). The resulting mixtures were inoculated in healthy mice (*n* = 5 for each group), and parasitaemia was evaluated five days post-infection. The mean values ± SEM of two independent experiments are reported for each group (Fig. 3). A significant reduction in the parasitemia of both *Wa*F17.12-KT^+^ (55.4%) and *Wa*F17.12-KT^+^ plus Ab (40.1%) groups was detected indicating that *Wa*F17.12-KT^+^ interferes with *Pb*GFP_CON_ erythrocytic stages and impairs the ability of the parasite to infect recipient mice. Interestingly, treatment with the monoclonal antibody mAbKT4 in part inhibited the effects of *Wa*F17.12-KT^+^ showing a reduction percentage that is lower with respect to the KT group. It is reasonable that a saturating concentration of the antibody could cause a complete block of *Wa*F17.12-KT activity.

**Fig. 4:**
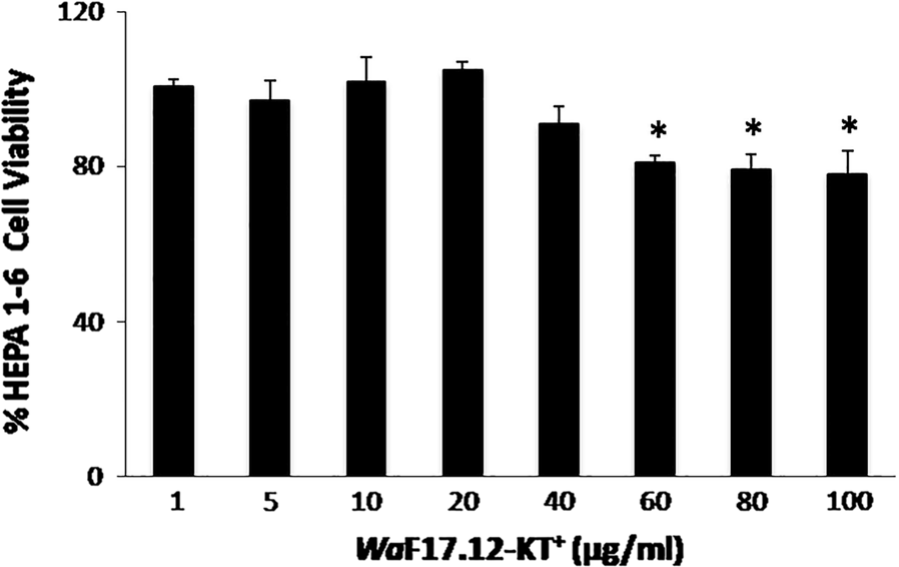
Safety test on murine cell line. HEPA 1–6 cells were cultured with different doses of *Wa*FA17.12-KT^+^ for 24 h. Cell viability was determined by MTT assay. Data shown are expressed as mean ± SD of three separate experiments; *P < 0.01 *vs* vehicle-treated cells

To evaluate the potential therapeutically use of *Wa*F17.12-KT, a preliminary test was performed using the murine cell line HEPA 1–6. A viability assay was carried out through a colorimetric MTT test. Cells were incubated with different doses of *Wa*F17.12-KT^+^ for 24 h (Fig. 4). Our data demonstrated that the treatment slightly affects cell viability only at the highest doses (60, 80 and 100 µg/ml KT), suggesting that KT can be considered safe on vertebrate cells. We carried out additional analysis using PI on HEPA 1–6 cells treated and not treated with KT, showing no cell damage at a dose of 100 μg/ml at 24 h of treatment (Additional file 1: Figure S1).

## Discussion

SC strategies propose the use of symbiotic microorganisms that interfere with the vector’s capability of blocking the transmission of malaria. In recent years, several bacteria and fungi have been indicated as good candidates for SC applications against malaria since they can inhibit the development of the parasite in wild mosquitoes. Direct or indirect anti-plasmodial mechanisms can be exerted by wild-type or recombinant microbial strains. In this context, the use of natural killer strains limits the introduction of genetically manipulated microorganisms into the environment. This aspect makes symbiotic strains of *W. anomalus* highly appealing for SC strategies, particularly after the discovery of their ability to produce KTs with anti-parasitic effects in the *Anopheles* midgut. In the present study, we demonstrated that a diet supplemented with activated *Wa*F17.12 cultures inhibits the development of the early sporogonic stages of *P. berghei* in *An. stephensi* (− 65.21%). Interestingly, the KT non-producer strain *Wa*UM3 also affected parasites (− 38.02%), likely by host immune system stimulation due to the massive presence of the yeast in the gut [28]. Nevertheless, the significantly higher effect of *Wa*F17.12 highlighted a direct mechanism due to the action of KT. We have used only the KT non-producer strain *Wa*UM3 as a negative control since a non-stimulated *Wa*F17.12 control group is not obtainable. In fact, it is possible to induce higher or lower KT production by cultural methods using growth media at different pH values but not KT free cultures [20].

These outcomes are consistent with the demonstration that, *in vitro*, only *Wa*F17.12 is able to prevent ookinete development (− 40%), supporting the hypothesis that the surface membrane damage is associated to *Wa*F17.12-KT β-glucanase activity, as previously reported by Valzano et al. [19].

Although *in vitro* and *in vivo* inhibition rates cannot be compared because of substantial differences between the two experimental systems, we could speculate that the interaction of *Wa*F17.12-KT with the parasite cell-wall carbohydrates may be more efficient in the midgut lumen.

We also investigated the development of later sporogonic stages in the mosquito fed with *Wa*F17.12, finding that there is no antagonist effect on oocysts and sporozoites. In fact, Cappelli et al. [20] reported that *Wa*F17.12-KT is released in the midgut lumen surrounding the epithelium, thus oocysts and sporozoites, differently from ookinetes, are not exposed to the toxin. Additionally, the 100-fold higher number of oocysts produced upon experimental infections with respect to natural conditions, could mask the anti-plasmodial effect of killer yeasts at the level of later sporogonic stages. In fact, the considered murine malaria system (*P. berghei*/*An. stephensi*/BALBc) is an experimental model optimized to have abundant infections in mosquitoes [29, 30]. To address this issue, additional tests using killer yeasts against human malaria parasites in naturally infected mosquitoes that develop no more than 5–10 oocysts [31], could be advisable. In fact, under these conditions *Wa*F17.12-KT could break down the vector capacity. Interestingly, yeasts are already used in traps because the smell produced by their fermentation is able to attract mosquitoes [32]. Thus, sugary preparations supplemented with *Wa*F17.12 cultures could be placed in traps to collect wild mosquitoes to be used for testing the anti-plasmodial effects on human malaria parasites. Further studies will assess symbiotic killer yeasts of anophelines as new candidates for innovative and safe SC-strategies of malaria.

The present study also provides evidence for the anti-plasmodial activity of *Wa*F17.12-KT on erythrocytic stages, as shown by the KT-mediated reduction of parasitatemia observed in experimentally infected mice.

Interestingly, *Wa*F17.12-KT was also active against parasite stages that develop in the vertebrate host, likely according to an antimicrobial mechanism of action based on glucanase activity. In fact, β-1,3-glucans are microbial cell wall components in charge of maintaining cell morphology and osmotic integrity [33]. Nevertheless, KT action could involve also different pathways, as a recent study reported the apoptotic activity of peptides mimicking KTs against the protozoan parasite *Toxoplasma gondii* [34]. However, further investigations on human malaria parasites are requested to assess the mechanism of action of KTs.

Regardless of the mechanisms of action, tests against a murine cell line showed that *Wa*F17.12-KT does not affect vertebrate cells.

## Conclusions

Overall, these results demonstrate the anti-plasmodial activity of *Wa*F17.12 on *P. berghei* sporogonic stages, showing that killer yeasts can potentially be used as safe and innovative tools against malaria. *Wa*F17.12 could be applicable for symbiotic control in the field, taking advantage of the fact that killer yeasts are easily releasable in mosquito feeding sites. Furthermore, the effect of *Wa*F17.12-KT against the erythrocytic stages suggest that yeast killer toxins can be used as novel antiparasitic drugs.

## Additional file

**Additional file 1: Figure S1.** KT treatment does not induce cell damage. PI staining on HEPA 1–6 cells treated and not treated with KT (100 μg/ml) analysed by cytofluorimeter. *Abbreviation*: MFI, mean fluorescence intensity.

## Data Availability

All data generated or analysed during this study are included in this published article and its additional file.

## Abbreviations

Ab: antibody
GFP: green fluorescent protein
KT: killer toxin
IFA: immunofluorescence assay
MTT: 3-(4,5-dimethylthiazol-2-yl)-2,5-diphenyltetrazolium bromide
PBS: phosphate-buffered saline
PI: propidium iodide
SC: symbiotic control
*Wa*: *Wickerhamomyces anomalus*
YPD: yeast peptone dextrose
