# Supplementary material for: Killer yeasts exert anti-plasmodial activities against the malaria parasite Plasmodium berghei in the vector mosquito Anopheles stephensi and in mice
Source: Parasit Vectors. 2019 Jul 2;12:329. doi: 10.1186/s13071-019-3587-4 (PMC6604151; doi:10.1186/s13071-019-3587-4)
Supplement: Supplementary file 1 — Additional file 1: Figure S1. KT treatment does not induce cell damage. PI staining on HEPA 1–6 cells treated and not treated with KT (100 μg/ml) analysed by cytofluorimeter. Abbreviation: MFI, mean fluorescence intensity. [file 13071_2019_3587_MOESM1_ESM.doc]

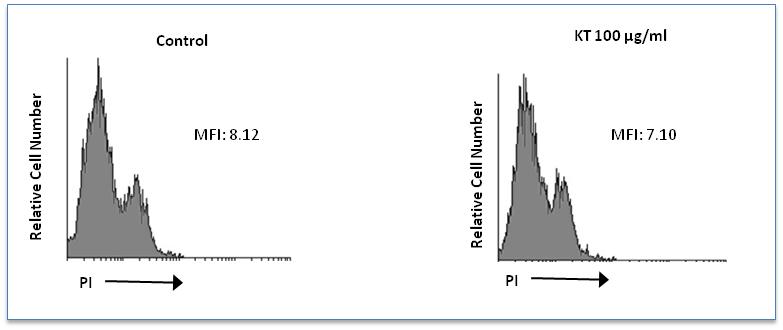


**Additional File 1: Figure S1**

**KT treatment does not induce cell damage**

PI staining on HEPA 1-6 cells treated and not treated with KT (100 μg/ml) analysed by cytofluorimeter. MFI= mean fluorescence intensity.
